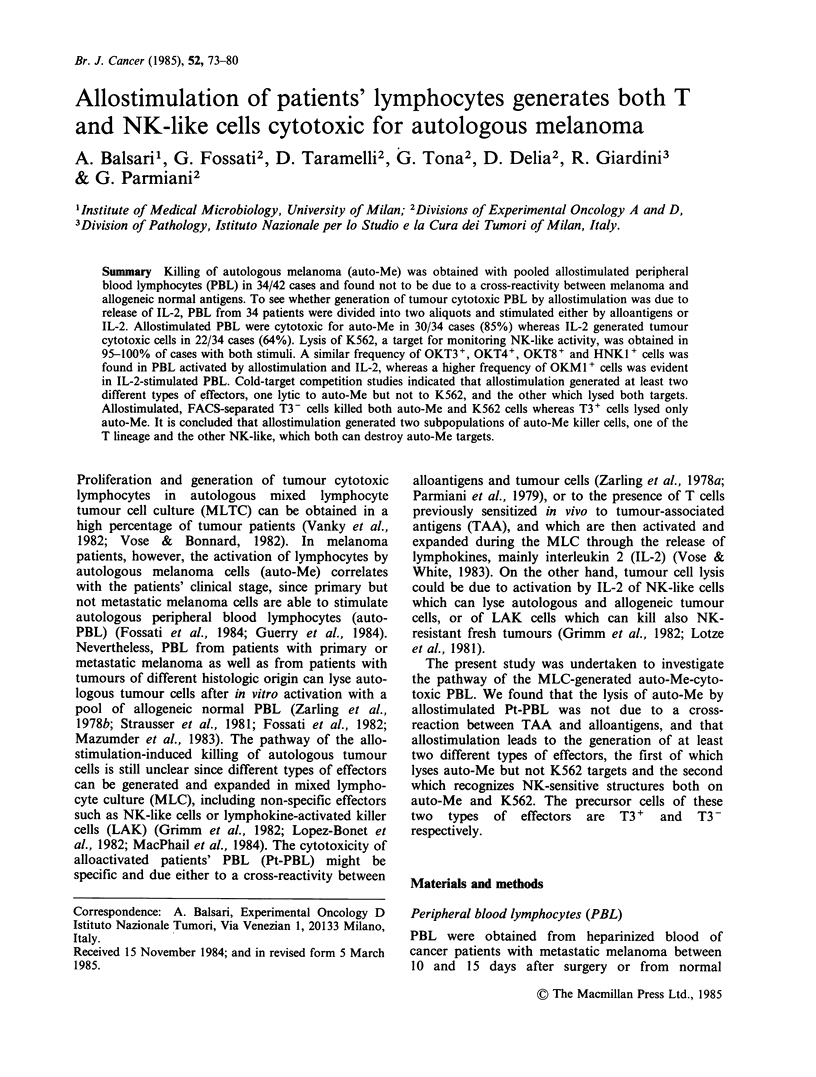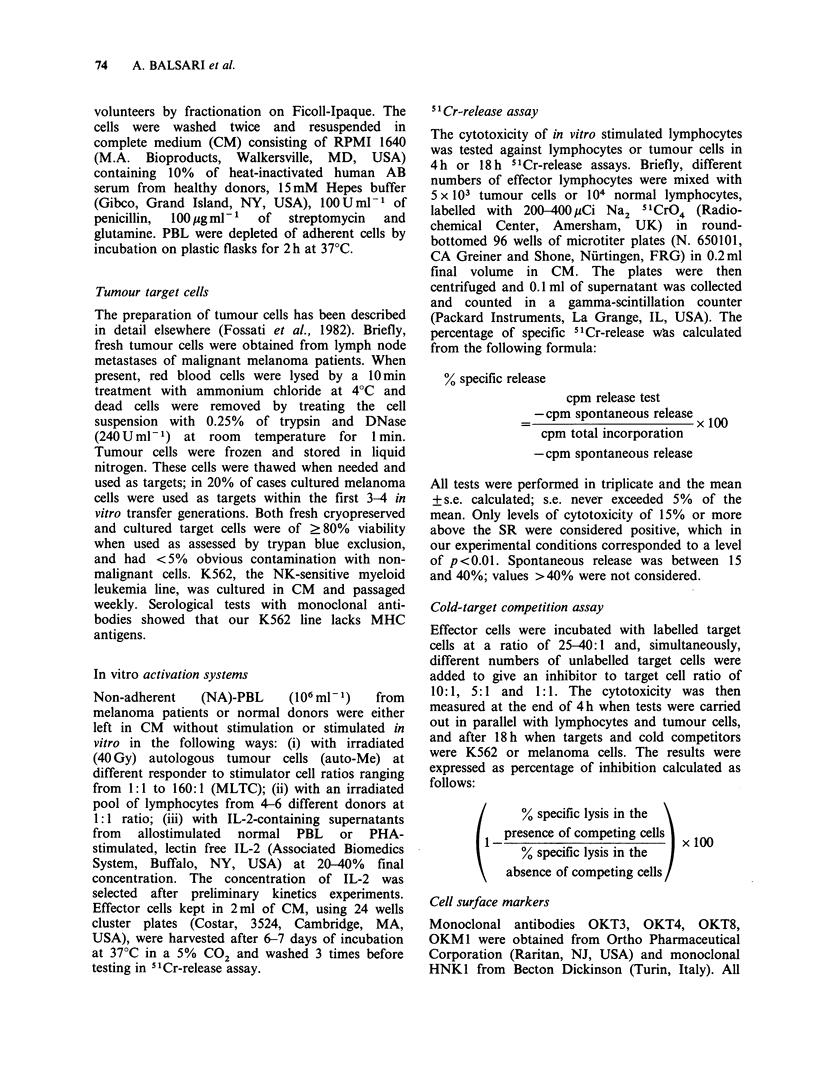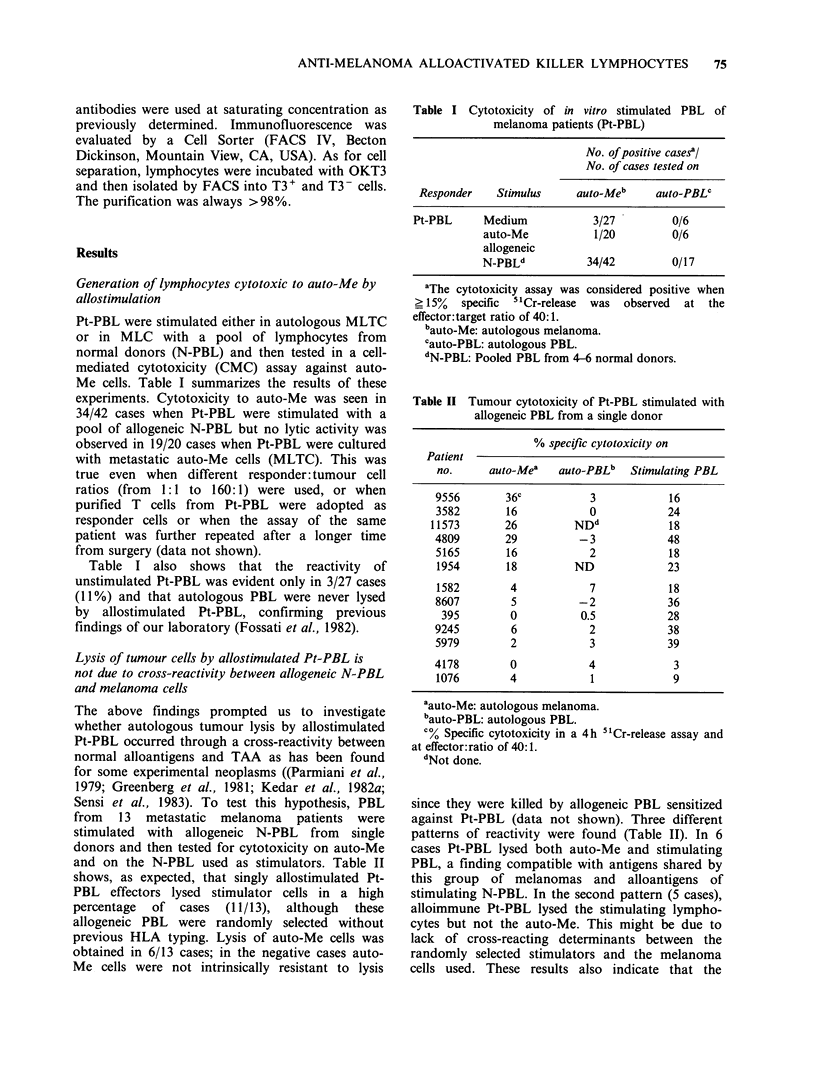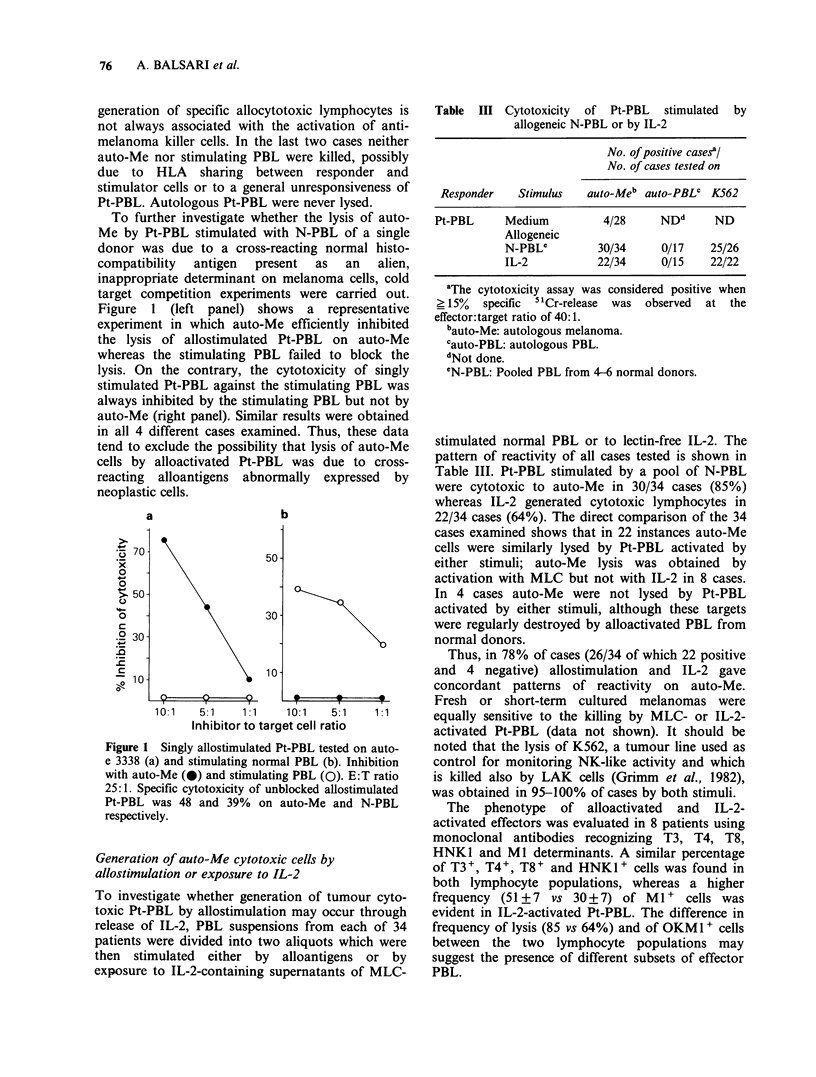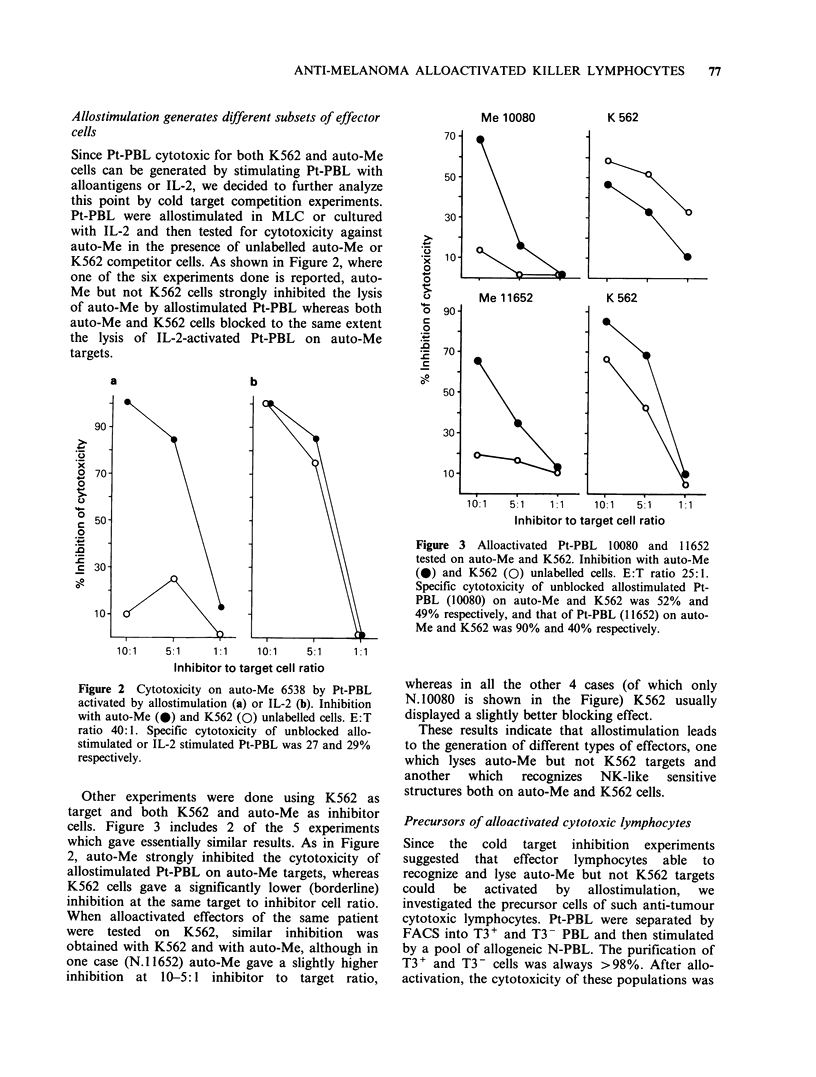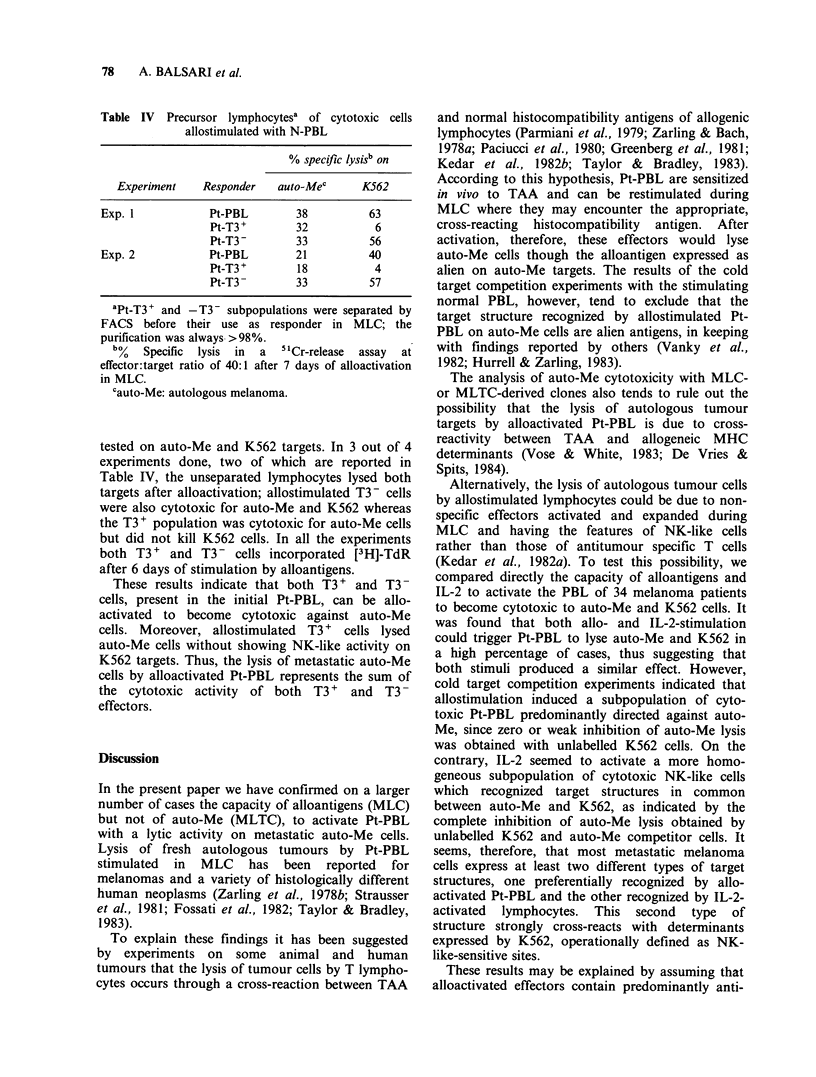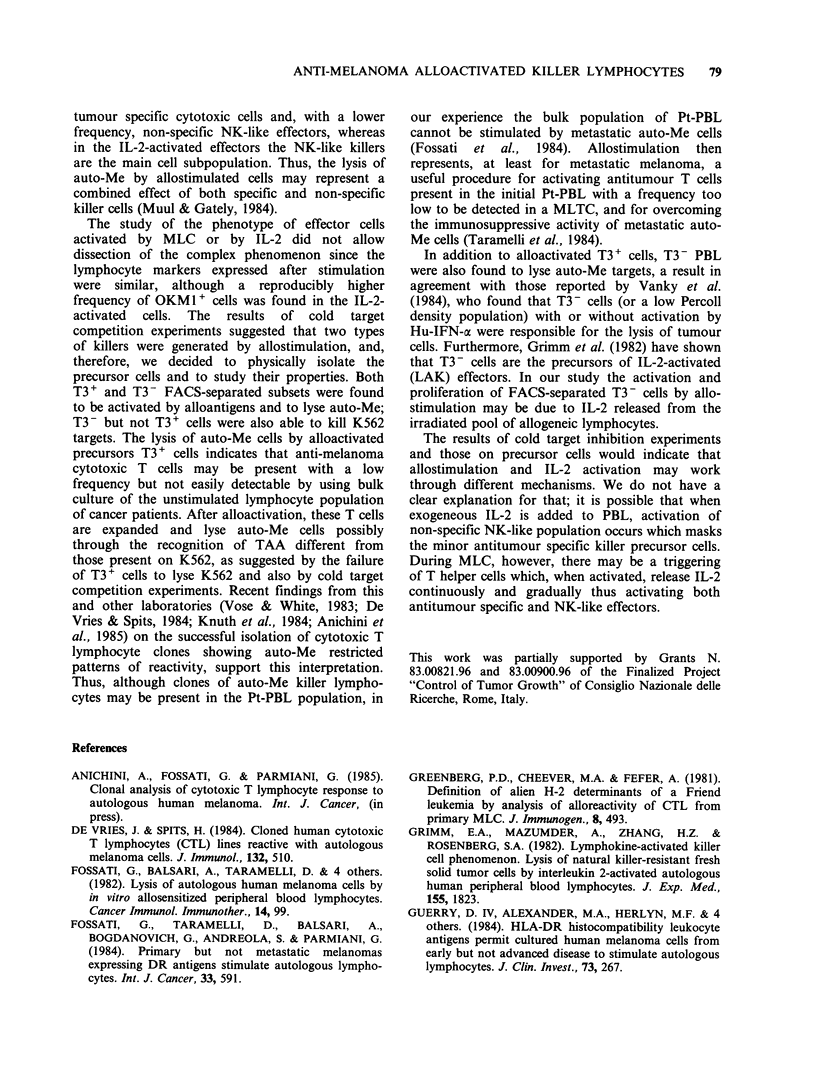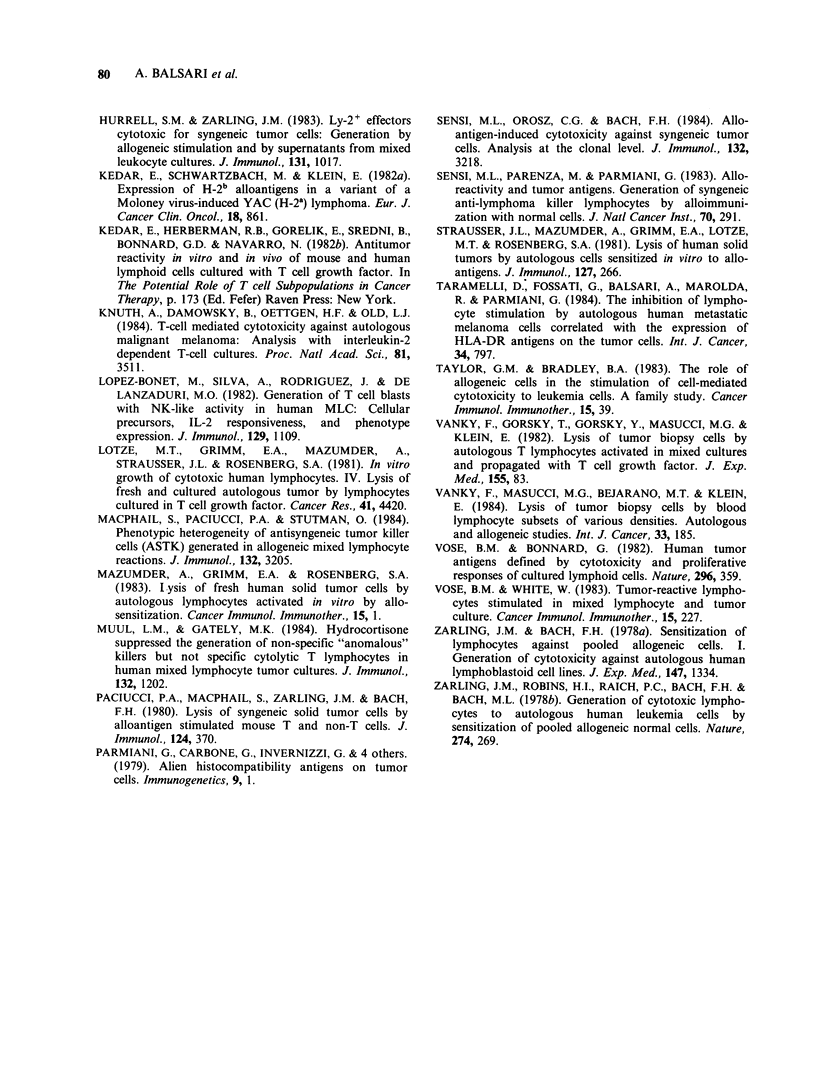# Allostimulation of patients' lymphocytes generates both T and NK-like cells cytotoxic for autologous melanoma.

**DOI:** 10.1038/bjc.1985.151

**Published:** 1985-07

**Authors:** A. Balsari, G. Fossati, D. Taramelli, G. Tona, D. Delia, R. Giardini, G. Parmiani

## Abstract

Killing of autologous melanoma (auto-Me) was obtained with pooled allostimulated peripheral blood lymphocytes (PBL) in 34/42 cases and found not to be due to a cross-reactivity between melanoma and allogeneic normal antigens. To see whether generation of tumour cytotoxic PBL by allostimulation was due to release of IL-2, PBL from 34 patients were divided into two aliquots and stimulated either by alloantigens or IL-2. Allostimulated PBL were cytotoxic for auto-Me in 30/34 cases (85%) whereas IL-2 generated tumour cytotoxic cells in 22/34 cases (64%). Lysis of K562, a target for monitoring NK-like activity, was obtained in 95-100% of cases with both stimuli. A similar frequency of OKT3+, OKT4+, OKT8+ and HNK1+ cells was found in PBL activated by allostimulation and IL-2, whereas a higher frequency of OKM1+ cells was evident in IL-2-stimulated PBL. Cold-target competition studies indicated that allostimulation generated at least two different types of effectors, one lytic to auto-Me but not to K562, and the other which lysed both targets. Allostimulated, FACS-separated T3- cells killed both auto-Me and K562 cells whereas T3+ cells lysed only auto-Me. It is concluded that allostimulation generated two subpopulations of auto-Me killer cells, one of the T lineage and the other NK-like, which both can destroy auto-Me targets.


*Br. J. Cancer* (1985), **52**, 73–80

### Allostimulation of patients' lymphocytes generates both T and NK-like cells cytotoxic for autologous melanoma

A. Balsari[1], G. Fossati[2], D. Taramelli[2], G. Tona[2], D. Delia[2], R. Giardini[3] & G. Parmiani[2]

[1]*Institute of Medical Microbiology, University of Milan;* [2]*Divisions of Experimental Oncology A and D,* [3]*Division of Pathology, Istituto Nazionale per lo Studio e la Cura dei Tumori of Milan, Italy.*

**Summary** Killing of autologous melanoma (auto-Me) was obtained with pooled allostimulated peripheral blood lymphocytes (PBL) in 34/42 cases and found not to be due to a cross-reactivity between melanoma and allogeneic normal antigens. To see whether generation of tumour cytotoxic PBL by allostimulation was due to release of IL-2, PBL from 34 patients were divided into two aliquots and stimulated either by alloantigens or IL-2. Allostimulated PBL were cytotoxic for auto-Me in 30/34 cases (85%) whereas IL-2 generated tumour cytotoxic cells in 22/34 cases (64%). Lysis of K562, a target for monitoring NK-like activity, was obtained in 95–100% of cases with both stimuli. A similar frequency of $OKT3^+$, $OKT4^+$, $OKT8^+$ and $HNK1^+$ cells was found in PBL activated by allostimulation and IL-2, whereas a higher frequency of $OKM1^+$ cells was evident in IL-2-stimulated PBL. Cold-target competition studies indicated that allostimulation generated at least two different types of effectors, one lytic to auto-Me but not to K562, and the other which lysed both targets. Allostimulated, FACS-separated $T3^-$ cells killed both auto-Me and K562 cells whereas $T3^+$ cells lysed only auto-Me. It is concluded that allostimulation generated two subpopulations of auto-Me killer cells, one of the T lineage and the other NK-like, which both can destroy auto-Me targets.

Proliferation and generation of tumour cytotoxic lymphocytes in autologous mixed lymphocyte tumour cell culture (MLTC) can be obtained in a high percentage of tumour patients (Vanky *et al.*, 1982; Vose & Bonnard, 1982). In melanoma patients, however, the activation of lymphocytes by autologous melanoma cells (auto-Me) correlates with the patients' clinical stage, since primary but not metastatic melanoma cells are able to stimulate autologous peripheral blood lymphocytes (auto-PBL) (Fossati *et al.*, 1984; Guerry *et al.*, 1984). Nevertheless, PBL from patients with primary or metastatic melanoma as well as from patients with tumours of different histologic origin can lyse autologous tumour cells after *in vitro* activation with a pool of allogeneic normal PBL (Zarling *et al.*, 1978*b*; Strausser *et al.*, 1981; Fossati *et al.*, 1982; Mazumder *et al.*, 1983). The pathway of the allostimulation-induced killing of autologous tumour cells is still unclear since different types of effectors can be generated and expanded in mixed lymphocyte culture (MLC), including non-specific effectors such as NK-like cells or lymphokine-activated killer cells (LAK) (Grimm *et al.*, 1982; Lopez-Bonet *et al.*, 1982; MacPhail *et al.*, 1984). The cytotoxicity of alloactivated patients' PBL (Pt-PBL) might be specific and due either to a cross-reactivity between alloantigens and tumour cells (Zarling *et al.*, 1978*a*; Parmiani *et al.*, 1979), or to the presence of T cells previously sensitized *in vivo* to tumour-associated antigens (TAA), and which are then activated and expanded during the MLC through the release of lymphokines, mainly interleukin 2 (IL-2) (Vose & White, 1983). On the other hand, tumour cell lysis could be due to activation by IL-2 of NK-like cells which can lyse autologous and allogeneic tumour cells, or of LAK cells which can kill also NK-resistant fresh tumours (Grimm *et al.*, 1982; Lotze *et al.*, 1981).

The present study was undertaken to investigate the pathway of the MLC-generated auto-Me-cytotoxic PBL. We found that the lysis of auto-Me by allostimulated Pt-PBL was not due to a cross-reaction between TAA and alloantigens, and that allostimulation leads to the generation of at least two different types of effectors, the first of which lyses auto-Me but not K562 targets and the second which recognizes NK-sensitive structures both on auto-Me and K562. The precursor cells of these two types of effectors are $T3^+$ and $T3^-$ respectively.

Correspondence: A. Balsari, Experimental Oncology D Istituto Nazionale Tumori, Via Venezian 1, 20133 Milano, Italy.

Received 15 November 1984; and in revised form 5 March 1985.

### Materials and methods

### *Peripheral blood lymphocytes (PBL)*

PBL were obtained from heparinized blood of cancer patients with metastatic melanoma between 10 and 15 days after surgery or from normal

© The Macmillan Press Ltd., 1985

volunteers by fractionation on Ficoll-Ipaque. The cells were washed twice and resuspended in complete medium (CM) consisting of RPMI 1640 (M.A. Bioproducts, Walkersville, MD, USA) containing 10% of heat-inactivated human AB serum from healthy donors, 15 mM Hepes buffer (Gibco, Grand Island, NY, USA), 100 U ml$^{-1}$ of penicillin, 100 $\mu$g ml$^{-1}$ of streptomycin and glutamine. PBL were depleted of adherent cells by incubation on plastic flasks for 2 h at 37°C.

### *Tumour target cells*

The preparation of tumour cells has been described in detail elsewhere (Fossati *et al.*, 1982). Briefly, fresh tumour cells were obtained from lymph node metastases of malignant melanoma patients. When present, red blood cells were lysed by a 10 min treatment with ammonium chloride at 4°C and dead cells were removed by treating the cell suspension with 0.25% of trypsin and DNase (240 U ml$^{-1}$) at room temperature for 1 min. Tumour cells were frozen and stored in liquid nitrogen. These cells were thawed when needed and used as targets; in 20% of cases cultured melanoma cells were used as targets within the first 3–4 *in vitro* transfer generations. Both fresh cryopreserved and cultured target cells were of $\geq$80% viability when used as assessed by trypan blue exclusion, and had <5% obvious contamination with non-malignant cells. K562, the NK-sensitive myeloid leukemia line, was cultured in CM and passaged weekly. Serological tests with monoclonal antibodies showed that our K562 line lacks MHC antigens.

### In vitro *activation systems*

Non-adherent (NA)-PBL (10$^6$ ml$^{-1}$) from melanoma patients or normal donors were either left in CM without stimulation or stimulated *in vitro* in the following ways: (i) with irradiated (40 Gy) autologous tumour cells (auto-Me) at different responder to stimulator cell ratios ranging from 1:1 to 160:1 (MLTC); (ii) with an irradiated pool of lymphocytes from 4–6 different donors at 1:1 ratio; (iii) with IL-2-containing supernatants from allostimulated normal PBL or PHA-stimulated, lectin free IL-2 (Associated Biomedics System, Buffalo, NY, USA) at 20–40% final concentration. The concentration of IL-2 was selected after preliminary kinetics experiments. Effector cells kept in 2 ml of CM, using 24 wells cluster plates (Costar, 3524, Cambridge, MA, USA), were harvested after 6–7 days of incubation at 37°C in a 5% $CO_2$ and washed 3 times before testing in $^{51}$Cr-release assay.

### *$^{51}$Cr-release assay*

The cytotoxicity of *in vitro* stimulated lymphocytes was tested against lymphocytes or tumour cells in 4 h or 18 h $^{51}$Cr-release assays. Briefly, different numbers of effector lymphocytes were mixed with $5\times10^3$ tumour cells or $10^4$ normal lymphocytes, labelled with 200–400 $\mu$Ci $Na_2$ $^{51}CrO_4$ (Radiochemical Center, Amersham, UK) in round-bottomed 96 wells of microtiter plates (N. 650101, CA Greiner and Shone, Nürtingen, FRG) in 0.2 ml final volume in CM. The plates were then centrifuged and 0.1 ml of supernatant was collected and counted in a gamma-scintillation counter (Packard Instruments, La Grange, IL, USA). The percentage of specific $^{51}$Cr-release was calculated from the following formula:

$$\%\text{ specific release} = \frac{\text{cpm release test} - \text{cpm spontaneous release}}{\text{cpm total incorporation} - \text{cpm spontaneous release}} \times 100$$

All tests were performed in triplicate and the mean $\pm$s.e. calculated; s.e. never exceeded 5% of the mean. Only levels of cytotoxicity of 15% or more above the SR were considered positive, which in our experimental conditions corresponded to a level of $p<0.01$. Spontaneous release was between 15 and 40%; values >40% were not considered.

### *Cold-target competition assay*

Effector cells were incubated with labelled target cells at a ratio of 25–40:1 and, simultaneously, different numbers of unlabelled target cells were added to give an inhibitor to target cell ratio of 10:1, 5:1 and 1:1. The cytotoxicity was then measured at the end of 4 h when tests were carried out in parallel with lymphocytes and tumour cells, and after 18 h when targets and cold competitors were K562 or melanoma cells. The results were expressed as percentage of inhibition calculated as follows:

$$\left(1 - \frac{\%\text{ specific lysis in the presence of competing cells}}{\%\text{ specific lysis in the absence of competing cells}}\right) \times 100$$

### *Cell surface markers*

Monoclonal antibodies OKT3, OKT4, OKT8, OKM1 were obtained from Ortho Pharmaceutical Corporation (Raritan, NJ, USA) and monoclonal HNK1 from Becton Dickinson (Turin, Italy). All antibodies were used at saturating concentration as previously determined. Immunofluorescence was evaluated by a Cell Sorter (FACS IV, Becton Dickinson, Mountain View, CA, USA). As for cell separation, lymphocytes were incubated with OKT3 and then isolated by FACS into $T3^+$ and $T3^-$ cells. The purification was always >98%.

### Results

### *Generation of lymphocytes cytotoxic to auto-Me by allostimulation*

Pt-PBL were stimulated either in autologous MLTC or in MLC with a pool of lymphocytes from normal donors (N-PBL) and then tested in a cell-mediated cytotoxicity (CMC) assay against auto-Me cells. Table I summarizes the results of these experiments. Cytotoxicity to auto-Me was seen in 34/42 cases when Pt-PBL were stimulated with a pool of allogeneic N-PBL but no lytic activity was observed in 19/20 cases when Pt-PBL were cultured with metastatic auto-Me cells (MLTC). This was true even when different responder:tumour cell ratios (from 1:1 to 160:1) were used, or when purified T cells from Pt-PBL were adopted as responder cells or when the assay of the same patient was further repeated after a longer time from surgery (data not shown).

Table I also shows that the reactivity of unstimulated Pt-PBL was evident only in 3/27 cases (11%) and that autologous PBL were never lysed by allostimulated Pt-PBL, confirming previous findings of our laboratory (Fossati *et al.*, 1982).

### *Lysis of tumour cells by allostimulated Pt-PBL is not due to cross-reactivity between allogeneic N-PBL and melanoma cells*

The above findings prompted us to investigate whether autologous tumour lysis by allostimulated Pt-PBL occurred through a cross-reactivity between normal alloantigens and TAA as has been found for some experimental neoplasms ((Parmiani *et al.*, 1979; Greenberg *et al.*, 1981; Kedar *et al.*, 1982*a*; Sensi *et al.*, 1983). To test this hypothesis, PBL from 13 metastatic melanoma patients were stimulated with allogeneic N-PBL from single donors and then tested for cytotoxicity on auto-Me and on the N-PBL used as stimulators. Table II shows, as expected, that singly allostimulated Pt-PBL effectors lysed stimulator cells in a high percentage of cases (11/13), although these allogeneic PBL were randomly selected without previous HLA typing. Lysis of auto-Me cells was obtained in 6/13 cases; in the negative cases auto-Me cells were not intrinsically resistant to lysis since they were killed by allogeneic PBL sensitized against Pt-PBL (data not shown). Three different patterns of reactivity were found (Table II). In 6 cases Pt-PBL lysed both auto-Me and stimulating PBL, a finding compatible with antigens shared by this group of melanomas and alloantigens of stimulating N-PBL. In the second pattern (5 cases), alloimmune Pt-PBL lysed the stimulating lymphocytes but not the auto-Me. This might be due to lack of cross-reacting determinants between the randomly selected stimulators and the melanoma cells used. These results also indicate that the

**Table I** Cytotoxicity of *in vitro* stimulated PBL of melanoma patients (Pt-PBL)

| *Responder* | *Stimulus* | *No. of positive cases[a]/ No. of cases tested on* auto-Me[b] | auto-PBL[c] |
|---|---|---|---|
| Pt-PBL | Medium | 3/27 | 0/6 |
| | auto-Me | 1/20 | 0/6 |
| | allogeneic N-PBL[d] | 34/42 | 0/17 |

[a]The cytotoxicity assay was considered positive when ≧15% specific $^{51}$Cr-release was observed at the effector:target ratio of 40:1.
[b]auto-Me: autologous melanoma.
[c]auto-PBL: autologous PBL.
[d]N-PBL: Pooled PBL from 4–6 normal donors.

**Table II** Tumour cytotoxicity of Pt-PBL stimulated with allogeneic PBL from a single donor

| *Patient no.* | *% specific cytotoxicity on* auto-Me[a] | auto-PBL[b] | Stimulating PBL |
|---|---|---|---|
| 9556 | 36[c] | 3 | 16 |
| 3582 | 16 | 0 | 24 |
| 11573 | 26 | ND[d] | 18 |
| 4809 | 29 | −3 | 48 |
| 5165 | 16 | 2 | 18 |
| 1954 | 18 | ND | 23 |
| 1582 | 4 | 7 | 18 |
| 8607 | 5 | −2 | 36 |
| 395 | 0 | 0.5 | 28 |
| 9245 | 6 | 2 | 38 |
| 5979 | 2 | 3 | 39 |
| 4178 | 0 | 4 | 3 |
| 1076 | 4 | 1 | 9 |

[a]auto-Me: autologous melanoma.
[b]auto-PBL: autologous PBL.
[c]% Specific cytotoxicity in a 4 h $^{51}$Cr-release assay and at effector:ratio of 40:1.
[d]Not done.

generation of specific allocytotoxic lymphocytes is not always associated with the activation of anti-melanoma killer cells. In the last two cases neither auto-Me nor stimulating PBL were killed, possibly due to HLA sharing between responder and stimulator cells or to a general unresponsiveness of Pt-PBL. Autologous Pt-PBL were never lysed.

To further investigate whether the lysis of auto-Me by Pt-PBL stimulated with N-PBL of a single donor was due to a cross-reacting normal histocompatibility antigen present as an alien, inappropriate determinant on melanoma cells, cold target competition experiments were carried out. Figure 1 (left panel) shows a representative experiment in which auto-Me efficiently inhibited the lysis of allostimulated Pt-PBL on auto-Me whereas the stimulating PBL failed to block the lysis. On the contrary, the cytotoxicity of singly stimulated Pt-PBL against the stimulating PBL was always inhibited by the stimulating PBL but not by auto-Me (right panel). Similar results were obtained in all 4 different cases examined. Thus, these data tend to exclude the possibility that lysis of auto-Me cells by alloactivated Pt-PBL was due to cross-reacting alloantigens abnormally expressed by neoplastic cells.

**Figure 1** Singly allostimulated Pt-PBL tested on auto-e 3338 (a) and stimulating normal PBL (b). Inhibition with auto-Me (●) and stimulating PBL (○). E:T ratio 25:1. Specific cytotoxicity of unblocked allostimulated Pt-PBL was 48 and 39% on auto-Me and N-PBL respectively.

### *Generation of auto-Me cytotoxic cells by allostimulation or exposure to IL-2*

To investigate whether generation of tumour cytotoxic Pt-PBL by allostimulation may occur through release of IL-2, PBL suspensions from each of 34 patients were divided into two aliquots which were then stimulated either by alloantigens or by exposure to IL-2-containing supernatants of MLC-stimulated normal PBL or to lectin-free IL-2. The pattern of reactivity of all cases tested is shown in Table III. Pt-PBL stimulated by a pool of N-PBL were cytotoxic to auto-Me in 30/34 cases (85%) whereas IL-2 generated cytotoxic lymphocytes in 22/34 cases (64%). The direct comparison of the 34 cases examined shows that in 22 instances auto-Me cells were similarly lysed by Pt-PBL activated by either stimuli; auto-Me lysis was obtained by activation with MLC but not with IL-2 in 8 cases. In 4 cases auto-Me were not lysed by Pt-PBL activated by either stimuli, although these targets were regularly destroyed by alloactivated PBL from normal donors.

**Table III** Cytotoxicity of Pt-PBL stimulated by allogeneic N-PBL or by IL-2

| | | *No. of positive cases[a]/ No. of cases tested on* | | |
|---|---|---|---|---|
| *Responder* | *Stimulus* | *auto-Me[b]* | *auto-PBL[c]* | *K562* |
| Pt-PBL | Medium | 4/28 | ND[d] | ND |
| | Allogeneic N-PBL[e] | 30/34 | 0/17 | 25/26 |
| | IL-2 | 22/34 | 0/15 | 22/22 |

[a]The cytotoxicity assay was considered positive when ≧15% specific $^{51}$Cr-release was observed at the effector:target ratio of 40:1.
[b]auto-Me: autologous melanoma.
[c]auto-PBL: autologous PBL.
[d]Not done.
[e]N-PBL: Pooled PBL from 4–6 normal donors.

Thus, in 78% of cases (26/34 of which 22 positive and 4 negative) allostimulation and IL-2 gave concordant patterns of reactivity on auto-Me. Fresh or short-term cultured melanomas were equally sensitive to the killing by MLC- or IL-2-activated Pt-PBL (data not shown). It should be noted that the lysis of K562, a tumour line used as control for monitoring NK-like activity and which is killed also by LAK cells (Grimm *et al.*, 1982), was obtained in 95–100% of cases by both stimuli.

The phenotype of alloactivated and IL-2-activated effectors was evaluated in 8 patients using monoclonal antibodies recognizing T3, T4, T8, HNK1 and M1 determinants. A similar percentage of $T3^+$, $T4^+$, $T8^+$ and $HNK1^+$ cells was found in both lymphocyte populations, whereas a higher frequency ($51 \pm 7$ *vs* $30 \pm 7$) of $M1^+$ cells was evident in IL-2-activated Pt-PBL. The difference in frequency of lysis (85 *vs* 64%) and of $OKM1^+$ cells between the two lymphocyte populations may suggest the presence of different subsets of effector PBL.

*Allostimulation generates different subsets of effector cells*

Since Pt-PBL cytotoxic for both K562 and auto-Me cells can be generated by stimulating Pt-PBL with alloantigens or IL-2, we decided to further analyze this point by cold target competition experiments. Pt-PBL were allostimulated in MLC or cultured with IL-2 and then tested for cytotoxicity against auto-Me in the presence of unlabelled auto-Me or K562 competitor cells. As shown in Figure 2, where one of the six experiments done is reported, auto-Me but not K562 cells strongly inhibited the lysis of auto-Me by allostimulated Pt-PBL whereas both auto-Me and K562 cells blocked to the same extent the lysis of IL-2-activated Pt-PBL on auto-Me targets.

**Figure 2** Cytotoxicity on auto-Me 6538 by Pt-PBL activated by allostimulation (**a**) or IL-2 (**b**). Inhibition with auto-Me (●) and K562 (○) unlabelled cells. E:T ratio 40:1. Specific cytotoxicity of unblocked allo-stimulated or IL-2 stimulated Pt-PBL was 27 and 29% respectively.

Other experiments were done using K562 as target and both K562 and auto-Me as inhibitor cells. Figure 3 includes 2 of the 5 experiments which gave essentially similar results. As in Figure 2, auto-Me strongly inhibited the cytotoxicity of allostimulated Pt-PBL on auto-Me targets, whereas K562 cells gave a significantly lower (borderline) inhibition at the same target to inhibitor cell ratio. When alloactivated effectors of the same patient were tested on K562, similar inhibition was obtained with K562 and with auto-Me, although in one case (N.11652) auto-Me gave a slightly higher inhibition at 10–5:1 inhibitor to target ratio,

**Figure 3** Alloactivated Pt-PBL 10080 and 11652 tested on auto-Me and K562. Inhibition with auto-Me (●) and K562 (○) unlabelled cells. E:T ratio 25:1. Specific cytotoxicity of unblocked allostimulated Pt-PBL (10080) on auto-Me and K562 was 52% and 49% respectively, and that of Pt-PBL (11652) on auto-Me and K562 was 90% and 40% respectively.

whereas in all the other 4 cases (of which only N.10080 is shown in the Figure) K562 usually displayed a slightly better blocking effect.

These results indicate that allostimulation leads to the generation of different types of effectors, one which lyses auto-Me but not K562 targets and another which recognizes NK-like sensitive structures both on auto-Me and K562 cells.

*Precursors of alloactivated cytotoxic lymphocytes*

Since the cold target inhibition experiments suggested that effector lymphocytes able to recognize and lyse auto-Me but not K562 targets could be activated by allostimulation, we investigated the precursor cells of such anti-tumour cytotoxic lymphocytes. Pt-PBL were separated by FACS into $T3^+$ and $T3^-$ PBL and then stimulated by a pool of allogeneic N-PBL. The purification of $T3^+$ and $T3^-$ cells was always >98%. After allo-activation, the cytotoxicity of these populations was

**Table IV** Precursor lymphocytes[a] of cytotoxic cells allostimulated with N-PBL

| | | *% specific lysis[b] on* | |
|---|---|---|---|
| *Experiment* | *Responder* | *auto-Me[c]* | *K562* |
| Exp. 1 | Pt-PBL | 38 | 63 |
| | Pt-T3$^+$ | 32 | 6 |
| | Pt-T3$^-$ | 33 | 56 |
| Exp. 2 | Pt-PBL | 21 | 40 |
| | Pt-T3$^+$ | 18 | 4 |
| | Pt-T3$^-$ | 33 | 57 |

[a]Pt-T3$^+$ and $-$T3$^-$ subpopulations were separated by FACS before their use as responder in MLC; the purification was always >98%.

[b]% Specific lysis in a $^{51}$Cr-release assay at effector:target ratio of 40:1 after 7 days of alloactivation in MLC.

[c]auto-Me: autologous melanoma.

tested on auto-Me and K562 targets. In 3 out of 4 experiments done, two of which are reported in Table IV, the unseparated lymphocytes lysed both targets after alloactivation; allostimulated T3$^-$ cells were also cytotoxic for auto-Me and K562 whereas the T3$^+$ population was cytotoxic for auto-Me cells but did not kill K562 cells. In all the experiments both T3$^+$ and T3$^-$ cells incorporated [$^3$H]-TdR after 6 days of stimulation by alloantigens.

These results indicate that both T3$^+$ and T3$^-$ cells, present in the initial Pt-PBL, can be alloactivated to become cytotoxic against auto-Me cells. Moreover, allostimulated T3$^+$ cells lysed auto-Me cells without showing NK-like activity on K562 targets. Thus, the lysis of metastatic auto-Me cells by alloactivated Pt-PBL represents the sum of the cytotoxic activity of both T3$^+$ and T3$^-$ effectors.

### Discussion

In the present paper we have confirmed on a larger number of cases the capacity of alloantigens (MLC) but not of auto-Me (MLTC), to activate Pt-PBL with a lytic activity on metastatic auto-Me cells. Lysis of fresh autologous tumours by Pt-PBL stimulated in MLC has been reported for melanomas and a variety of histologically different human neoplasms (Zarling *et al.*, 1978*b*; Strausser *et al.*, 1981; Fossati *et al.*, 1982; Taylor & Bradley, 1983).

To explain these findings it has been suggested by experiments on some animal and human tumours that the lysis of tumour cells by T lymphocytes occurs through a cross-reaction between TAA and normal histocompatibility antigens of allogenic lymphocytes (Parmiani *et al.*, 1979; Zarling & Bach, 1978*a*; Paciucci *et al.*, 1980; Greenberg *et al.*, 1981; Kedar *et al.*, 1982*b*; Taylor & Bradley, 1983). According to this hypothesis, Pt-PBL are sensitized *in vivo* to TAA and can be restimulated during MLC where they may encounter the appropriate, cross-reacting histocompatibility antigen. After activation, therefore, these effectors would lyse auto-Me cells though the alloantigen expressed as alien on auto-Me targets. The results of the cold target competition experiments with the stimulating normal PBL, however, tend to exclude that the target structure recognized by allostimulated Pt-PBL on auto-Me cells are alien antigens, in keeping with findings reported by others (Vanky *et al.*, 1982; Hurrell & Zarling, 1983).

The analysis of auto-Me cytotoxicity with MLC- or MLTC-derived clones also tends to rule out the possibility that the lysis of autologous tumour targets by alloactivated Pt-PBL is due to cross-reactivity between TAA and allogeneic MHC determinants (Vose & White, 1983; De Vries & Spits, 1984).

Alternatively, the lysis of autologous tumour cells by allostimulated lymphocytes could be due to non-specific effectors activated and expanded during MLC and having the features of NK-like cells rather than those of antitumour specific T cells (Kedar *et al.*, 1982*a*). To test this possibility, we compared directly the capacity of alloantigens and IL-2 to activate the PBL of 34 melanoma patients to become cytotoxic to auto-Me and K562 cells. It was found that both allo- and IL-2-stimulation could trigger Pt-PBL to lyse auto-Me and K562 in a high percentage of cases, thus suggesting that both stimuli produced a similar effect. However, cold target competition experiments indicated that allostimulation induced a subpopulation of cytotoxic Pt-PBL predominantly directed against auto-Me, since zero or weak inhibition of auto-Me lysis was obtained with unlabelled K562 cells. On the contrary, IL-2 seemed to activate a more homogeneous subpopulation of cytotoxic NK-like cells which recognized target structures in common between auto-Me and K562, as indicated by the complete inhibition of auto-Me lysis obtained by unlabelled K562 and auto-Me competitor cells. It seems, therefore, that most metastatic melanoma cells express at least two different types of target structures, one preferentially recognized by alloactivated Pt-PBL and the other recognized by IL-2-activated lymphocytes. This second type of structure strongly cross-reacts with determinants expressed by K562, operationally defined as NK-like-sensitive sites.

These results may be explained by assuming that alloactivated effectors contain predominantly antitumour specific cytotoxic cells and, with a lower frequency, non-specific NK-like effectors, whereas in the IL-2-activated effectors the NK-like killers are the main cell subpopulation. Thus, the lysis of auto-Me by allostimulated cells may represent a combined effect of both specific and non-specific killer cells (Muul & Gately, 1984).

The study of the phenotype of effector cells activated by MLC or by IL-2 did not allow dissection of the complex phenomenon since the lymphocyte markers expressed after stimulation were similar, although a reproducibly higher frequency of $OKM1^+$ cells was found in the IL-2-activated cells. The results of cold target competition experiments suggested that two types of killers were generated by allostimulation, and, therefore, we decided to physically isolate the precursor cells and to study their properties. Both $T3^+$ and $T3^-$ FACS-separated subsets were found to be activated by alloantigens and to lyse auto-Me; $T3^-$ but not $T3^+$ cells were also able to kill K562 targets. The lysis of auto-Me cells by alloactivated precursors $T3^+$ cells indicates that anti-melanoma cytotoxic T cells may be present with a low frequency but not easily detectable by using bulk culture of the unstimulated lymphocyte population of cancer patients. After alloactivation, these T cells are expanded and lyse auto-Me cells possibly through the recognition of TAA different from those present on K562, as suggested by the failure of $T3^+$ cells to lyse K562 and also by cold target competition experiments. Recent findings from this and other laboratories (Vose & White, 1983; De Vries & Spits, 1984; Knuth *et al.*, 1984; Anichini *et al.*, 1985) on the successful isolation of cytotoxic T lymphocyte clones showing auto-Me restricted patterns of reactivity, support this interpretation. Thus, although clones of auto-Me killer lymphocytes may be present in the Pt-PBL population, in our experience the bulk population of Pt-PBL cannot be stimulated by metastatic auto-Me cells (Fossati *et al.*, 1984). Allostimulation then represents, at least for metastatic melanoma, a useful procedure for activating antitumour T cells present in the initial Pt-PBL with a frequency too low to be detected in a MLTC, and for overcoming the immunosuppressive activity of metastatic auto-Me cells (Taramelli *et al.*, 1984).

In addition to alloactivated $T3^+$ cells, $T3^-$ PBL were also found to lyse auto-Me targets, a result in agreement with those reported by Vanky *et al.* (1984), who found that $T3^-$ cells (or a low Percoll density population) with or without activation by Hu-IFN-$\alpha$ were responsible for the lysis of tumour cells. Furthermore, Grimm *et al.* (1982) have shown that $T3^-$ cells are the precursors of IL-2-activated (LAK) effectors. In our study the activation and proliferation of FACS-separated $T3^-$ cells by allostimulation may be due to IL-2 released from the irradiated pool of allogeneic lymphocytes.

The results of cold target inhibition experiments and those on precursor cells would indicate that allostimulation and IL-2 activation may work through different mechanisms. We do not have a clear explanation for that; it is possible that when exogeneous IL-2 is added to PBL, activation of non-specific NK-like population occurs which masks the minor antitumour specific killer precursor cells. During MLC, however, there may be a triggering of T helper cells which, when activated, release IL-2 continuously and gradually thus activating both antitumour specific and NK-like effectors.

This work was partially supported by Grants N. 83.00821.96 and 83.00900.96 of the Finalized Project "Control of Tumor Growth" of Consiglio Nazionale delle Ricerche, Rome, Italy.

### References

ANICHINI, A., FOSSATI, G. & PARMIANI, G. (1985). Clonal analysis of cytotoxic T lymphocyte response to autologous human melanoma. *Int. J. Cancer*, (in press).

DE VRIES, J. & SPITS, H. (1984). Cloned human cytotoxic T lymphocytes (CTL) lines reactive with autologous melanoma cells. *J. Immunol.*, **132**, 510.

FOSSATI, G., BALSARI, A., TARAMELLI, D. & 4 others. (1982). Lysis of autologous human melanoma cells by *in vitro* allosensitized peripheral blood lymphocytes. *Cancer Immunol. Immunother.*, **14**, 99.

FOSSATI, G., TARAMELLI, D., BALSARI, A., BOGDANOVICH, G., ANDREOLA, S. & PARMIANI, G. (1984). Primary but not metastatic melanomas expressing DR antigens stimulate autologous lymphocytes. *Int. J. Cancer*, **33**, 591.

GREENBERG, P.D., CHEEVER, M.A. & FEFER, A. (1981). Definition of alien H-2 determinants of a Friend leukemia by analysis of alloreactivity of CTL from primary MLC. *J. Immunogen.*, **8**, 493.

GRIMM, E.A., MAZUMDER, A., ZHANG, H.Z. & ROSENBERG, S.A. (1982). Lymphokine-activated killer cell phenomenon. Lysis of natural killer-resistant fresh solid tumor cells by interleukin 2-activated autologous human peripheral blood lymphocytes. *J. Exp. Med.*, **155**, 1823.

GUERRY, D. IV, ALEXANDER, M.A., HERLYN, M.F. & 4 others. (1984). HLA-DR histocompatibility leukocyte antigens permit cultured human melanoma cells from early but not advanced disease to stimulate autologous lymphocytes. *J. Clin. Invest.*, **73**, 267.

HURRELL, S.M. & ZARLING, J.M. (1983). Ly-$2^+$ effectors cytotoxic for syngeneic tumor cells: Generation by allogeneic stimulation and by supernatants from mixed leukocyte cultures. *J. Immunol.*, **131**, 1017.

KEDAR, E., SCHWARTZBACH, M. & KLEIN, E. (1982*a*). Expression of H-$2^b$ alloantigens in a variant of a Moloney virus-induced YAC (H-$2^a$) lymphoma. *Eur. J. Cancer Clin. Oncol.*, **18**, 861.

KEDAR, E., HERBERMAN, R.B., GORELIK, E., SREDNI, B., BONNARD, G.D. & NAVARRO, N. (1982*b*). Antitumor reactivity *in vitro* and *in vivo* of mouse and human lymphoid cells cultured with T cell growth factor. In *The Potential Role of T cell Subpopulations in Cancer Therapy*, p. 173 (Ed. Fefer) Raven Press: New York.

KNUTH, A., DAMOWSKY, B., OETTGEN, H.F. & OLD, L.J. (1984). T-cell mediated cytotoxicity against autologous malignant melanoma: Analysis with interleukin-2 dependent T-cell cultures. *Proc. Natl Acad. Sci.*, **81**, 3511.

LOPEZ-BONET, M., SILVA, A., RODRIGUEZ, J. & DE LANZADURI, M.O. (1982). Generation of T cell blasts with NK-like activity in human MLC: Cellular precursors, IL-2 responsiveness, and phenotype expression. *J. Immunol.*, **129**, 1109.

LOTZE, M.T., GRIMM, E.A., MAZUMDER, A., STRAUSSER, J.L. & ROSENBERG, S.A. (1981). *In vitro* growth of cytotoxic human lymphocytes. IV. Lysis of fresh and cultured autologous tumor by lymphocytes cultured in T cell growth factor. *Cancer Res.*, **41**, 4420.

MACPHAIL, S., PACIUCCI, P.A. & STUTMAN, O. (1984). Phenotypic heterogeneity of antisyngeneic tumor killer cells (ASTK) generated in allogeneic mixed lymphocyte reactions. *J. Immunol.*, **132**, 3205.

MAZUMDER, A., GRIMM, E.A. & ROSENBERG, S.A. (1983). Lysis of fresh human solid tumor cells by autologous lymphocytes activated *in vitro* by allosensitization. *Cancer Immunol. Immunother.*, **15**, 1.

MUUL, L.M., & GATELY, M.K. (1984). Hydrocortisone suppressed the generation of non-specific "anomalous" killers but not specific cytolytic T lymphocytes in human mixed lymphocyte tumor cultures. *J. Immunol.*, **132**, 1202.

PACIUCCI, P.A., MACPHAIL, S., ZARLING, J.M. & BACH, F.H. (1980). Lysis of syngeneic solid tumor cells by alloantigen stimulated mouse T and non-T cells. *J. Immunol.*, **124**, 370.

PARMIANI, G., CARBONE, G., INVERNIZZI, G. & 4 others. (1979). Alien histocompatibility antigens on tumor cells. *Immunogenetics*, **9**, 1.

SENSI, M.L., OROSZ, C.G. & BACH, F.H. (1984). Alloantigen-induced cytotoxicity against syngeneic tumor cells. Analysis at the clonal level. *J. Immunol.*, **132**, 3218.

SENSI, M.L., PARENZA, M. & PARMIANI, G. (1983). Alloreactivity and tumor antigens. Generation of syngeneic anti-lymphoma killer lymphocytes by alloimmunization with normal cells. *J. Natl Cancer Inst.*, **70**, 291.

STRAUSSER, J.L., MAZUMDER, A., GRIMM, E.A., LOTZE, M.T. & ROSENBERG, S.A. (1981). Lysis of human solid tumors by autologous cells sensitized *in vitro* to alloantigens. *J. Immunol.*, **127**, 266.

TARAMELLI, D., FOSSATI, G., BALSARI, A., MAROLDA, R. & PARMIANI, G. (1984). The inhibition of lymphocyte stimulation by autologous human metastatic melanoma cells correlated with the expression of HLA-DR antigens on the tumor cells. *Int. J. Cancer*, **34**, 797.

TAYLOR, G.M. & BRADLEY, B.A. (1983). The role of allogeneic cells in the stimulation of cell-mediated cytotoxicity to leukemia cells. A family study. *Cancer Immunol. Immunother.*, **15**, 39.

VANKY, F., GORSKY, T., GORSKY, Y., MASUCCI, M.G. & KLEIN, E. (1982). Lysis of tumor biopsy cells by autologous T lymphocytes activated in mixed cultures and propagated with T cell growth factor. *J. Exp. Med.*, **155**, 83.

VANKY, F., MASUCCI, M.G., BEJARANO, M.T. & KLEIN, E. (1984). Lysis of tumor biopsy cells by blood lymphocyte subsets of various densities. Autologous and allogeneic studies. *Int. J. Cancer*, **33**, 185.

VOSE, B.M. & BONNARD, G. (1982). Human tumor antigens defined by cytotoxicity and proliferative responses of cultured lymphoid cells. *Nature*, **296**, 359.

VOSE, B.M. & WHITE, W. (1983). Tumor-reactive lymphocytes stimulated in mixed lymphocyte and tumor culture. *Cancer Immunol. Immunother.*, **15**, 227.

ZARLING, J.M. & BACH, F.H. (1978*a*). Sensitization of lymphocytes against pooled allogeneic cells. I. Generation of cytotoxicity against autologous human lymphoblastoid cell lines. *J. Exp. Med.*, **147**, 1334.

ZARLING, J.M., ROBINS, H.I., RAICH, P.C., BACH, F.H. & BACH, M.L. (1978*b*). Generation of cytotoxic lymphocytes to autologous human leukemia cells by sensitization of pooled allogeneic normal cells. *Nature*, **274**, 269.